# Shadows of the mind: history of neurotrauma in the 19^th^ century

**DOI:** 10.25122/jml-2024-1001

**Published:** 2024-01

**Authors:** Stefana-Andrada Dobran, Livia Livint Popa, Dafin Muresanu

**Affiliations:** 1 RoNeuro Institute for Neurological Research and Diagnostic, Cluj-Napoca, Romania; 2 Department of Neuroscience, Iuliu Hatieganu University of Medicine and Pharmacy, Cluj-Napoca, Romania

## INTRODUCTION

Neurotrauma represents a ubiquitous yet understudied domain in neurosciences. A condition as old as time, it offers a glance into the nature of humankind, revealing a contrasting picture of violence, healing, scientific practices, and mystical beliefs. As innovations and breakthroughs in the 19^th^ century laid the groundwork for advancements in treatment, diagnostic techniques, and patient care today, understanding the history of neurotrauma not only helps us appreciate the development of modern knowledge and medical practice, but also offers a psychological, philosophical, and anthropological perspective of the human mind.

Limited medical knowledge and diagnostic tools as well as scarcity of therapeutic options represented dire challenges in managing neurotrauma. During this period, traumatic injuries were often associated with wars, industrial accidents, transportation mishaps, and interpersonal violence, with scientists and doctors possessing limited understanding of the long-term effects. Up to this time, medical interventions were largely limited to supportive care, including wound management, pain relief, and basic nursing, and surgical interventions carried significant risks due to a limited understanding of anatomy, infection control, and anesthesia [1,2].

**Figure 1:**
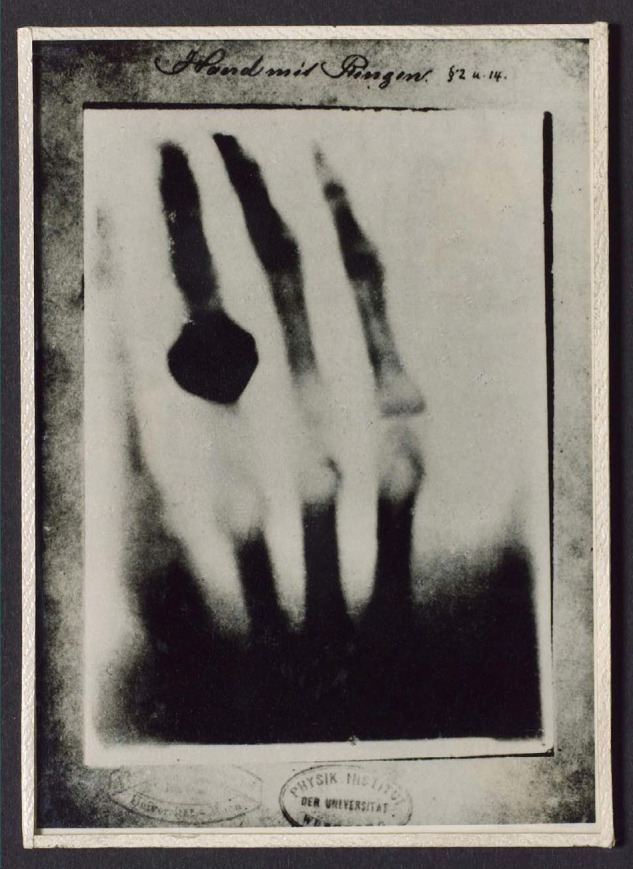
Copy of photograph of a radiograph of a hand Science Museum Group Collection © The Board of Trustees of the Science Museum. Available from: https://collection.sciencemuseumgroup.org.uk/objects/co134691/photograph-of-a-radiograph-of-hand-taken-by-wilhelm-conrad-rontgen-germany-1895-photograph

The 19^th^ century witnessed several developments, including the clinical examination based on neurological localization. The Napoleonic Wars led to innovation and breakthroughs in the science of cranial injuries, with notable contributions, including those of Baron Larrey (1766-1842) in France, in the domain of ipsilateral disorders, and George Guthrie (1788-1856) in Britain, on the evacuation of epidural hematoma and abscess [1]. Other advancements included the first mention of anisocoria, by Jonathan Hutchinson, the discovery of medical imaging by Roentgen (1895) (Figure 1), the development of asepsis and antisepsis, as well as the introduction of anesthesia; all leading to better management of spinal and cranial injuries [1-3]. Moreover, Bayle (1826) proposed the theory of chronic rebleeding as a potential pathogenesis for chronic subdural hematoma, while Hulke (1883) implemented the first successful neurosurgical treatment of subdural hematoma [4].

Neuroanatomy emerged as a distinct field of study, and significant advances were made in neurophysiology during that time. Pierre Paul Broca (1824–1880), Carl Wernicke (1848–1905), Korbinian Brodmann (1868–1918), and Santiago Ramón y Cajal (1852-1934) contributed to the understanding of the nervous system structure and functions, bringing significant contributions in understanding language, the organization of the cerebral cortex and its functional specialization through cytoarchitectonic mapping, and neuron theory [5].

## THE ENIGMA OF PHINEAS GAGE: A LANDMARK IN NEUROSCIENCE HISTORY

**Figure 2:**
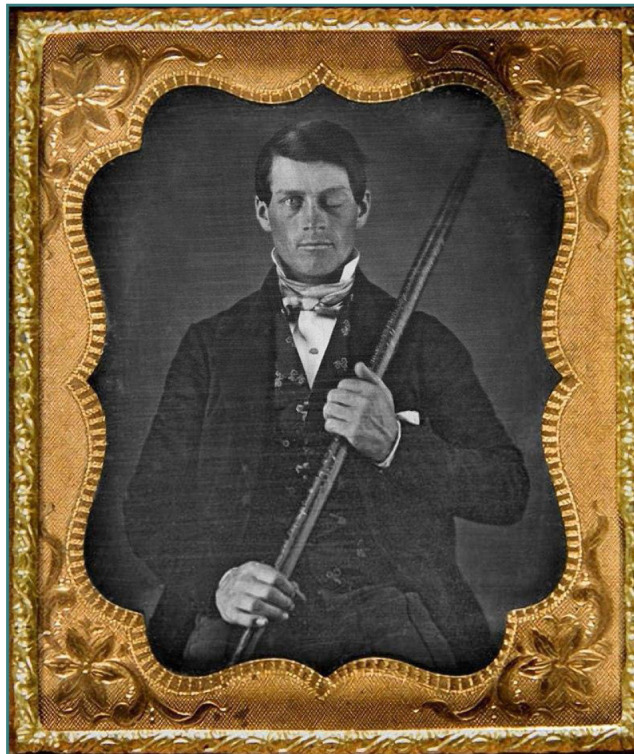
Portrait of Phineas P. Gage (1823–1860). Originally from the collection of Jack and Beverly Wilgus, and now in the Warren Anatomical Museum, Harvard Medical School (2017)

A striking case of neurotrauma that marked the 19^th^ century was that of Phineas P. Gage (1848) (Figure 2), a 25-year-old construction worker injured when an explosion propelled an iron bar through his left cheek, affecting his left frontal lobe and causing severe injuries. The injury was complicated by an infection that caused a state of semi-consciousness for a month. Doctor John Harlow treated Gage and helped him regain consciousness. Although Gage remained blind in one eye and experienced left facial weaknesses, he did not seem to present neurological deficits. However, surprising behavioral changes appeared later, ultimately described as frontal lobe syndrome [6-9].

Dr Harlow observed partial recovery post-injury, with mental abilities being slightly affected. His accident led to one of the first reports of psychiatric symptomatology post-traumatic brain injury (TBI). Although his memory did not seem to be affected, his personality changes were striking, leading to job loss and compromised image: he became negligent, capricious, childish, profane, and unable to take responsibility “... A child in his intellectual capacity and manifestations, he has the animal passions of a strong man…” [7,8,10]. The change from a previously responsible and well-adapted adult was striking for the ones around him. Gage then traveled with the iron throughout New England, as part of the Circus, and, 12 years after the incident, died of status epilepticus.

The ‘American Crowbar Case’ represented a landmark in the theory of cerebral localization. Despite the relevance of the findings, the case was ignored for several years, with people not being aware of Harlow’s 1868 report describing the accident and medical treatment until Dr. David Ferrier had a distinct interest in the localization of cerebral function [6]. He found that the effects of damage to the cortex (cortical lesions) depend on both the localization and nature of the damage. Gage’s experience highlighted Dr Ferrier’s findings: the prefrontal cortex was a functional brain area that impacts behavior, attention, and intelligence [6,7].

The case of Phineas Gage is one of the most influential stories in neuroscience, even though the conclusions and interpretations are incomplete. Further research used modern neuroimaging techniques, modeling several trajectories of the iron rod for the site of injury, after analyzing, measuring, and photographing the skull at Harvard Medical School Museum, to better interpret Gage’s lesion and the relationship to his behavior [10].

## CAPTURING MOTION: THE LEGACY OF EADWEARD MUYBRIDGE

**Figure 3:**
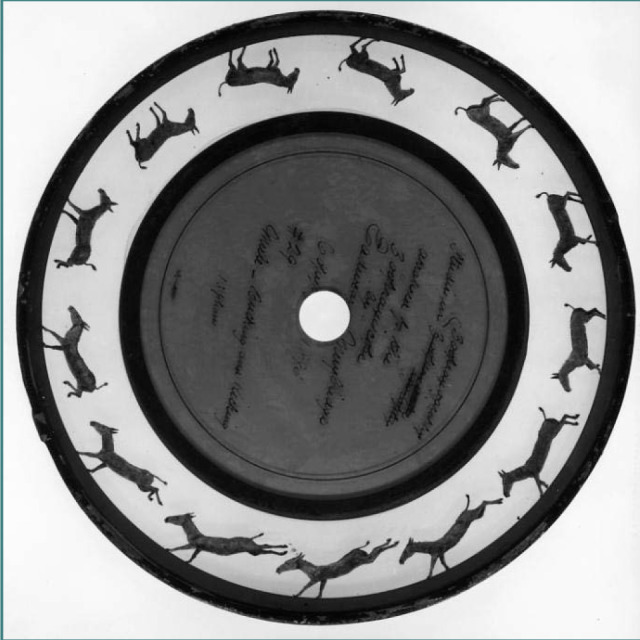
Picture of a zoopraxiscope disc, circa 1893 by Eadweard Muybridge and Erwin F. Faber. Retrieved from: https://commons.wikimedia.org/wiki/File:Zoopraxiscope_16485u.jpg

Another notable story of the 19^th^ century, parallel to that of Phineas Gage, was that of the Anglo-American photographer Eadweard Muybridge (1830-1904), a pioneer in photography and the inventor of the zoopraxiscope (predecessor of motion pictures) (Figure 3). Besides his sparkling career in photography, marked by two original patents in photographic technology, he also made significant contributions to art and neurology. His first experiments with motion photography aimed to prove that during the gait of a trotting horse, all four legs are off the ground simultaneously. Although contested by critics, he lectured on animal locomotion throughout the US and Europe [11-13].

Muybridge began his professional path as a bookseller. After sustaining a significant brain injury due to a vehicle accident, his life changed forever. He was treated for three months, yet the accident altered his abilities as well as his behavior, changing his life forever. Muybridge did not seem to remember his accident and suffered from impaired smell and taste, as well as post-recovery individual images in each eye [13].

Although there was no clear assessment due to the lack of imaging and neuropsychological tools at that time, his symptoms suggested frontal lobe injury or injury to the orbitofrontal cortex. His behavior altered, becoming aggressive, possessive, unstable, and presenting obsessive-compulsive traits. He changed his name several times and even carried out a murder of which he was acquitted; a staggering contrast from his previously friendly demeanor. At the recommendation of his doctor, he embraced photography as a profession. Developing a passion for observing animal and human locomotion, his work promoted progress in biomechanics and neurology, by analyzing sequential motion photographs using multiple cameras and studying time through a deconstruction of photography and motion pictures. The theories suggest his behavioral changes resemble certain aspects observed in the manifestation of frontotemporal dementia, which can sometimes lead to the development of artistic expression in affected individuals through disinhibited expression of emotions through art. This phenomenon of enhanced function in one brain area due to functional loss in another is known as *paradoxical functional facilitation* [13].

A daring artist, photographer, inventor, and lecturer who influenced the photography and movie industry, Muybridge’s case represents a fascinating insight into the complexities of the human mind. His fascination with human and animal motion has helped neurologists interested in moving disorders. Whether or not partly resulting from the injury, his genius was undeniable.

## CONCLUSIONS

Overall, the 19^th^ century marked a period of significant progress in the history of neurotrauma. Understanding neurological function and brain localization, as well as shifting the classification paradigm regarding head injuries has paved the way towards more precise diagnoses and improved outcomes. However, while significant strides were made in foundational neuroscience, managing TBI remains challenging. In a time marked by the developments of the Industrial Revolution, political conflicts, and scientific advancements that forever influenced the trajectory of medicine and highly improved survival, reflecting on some of the most significant progress and personalities in the history of neurotrauma in the 19^th^ century offers an enhanced appreciation for those who directly or indirectly shaped the field.

Despite extended efforts and even considering the significant body of literature, the domain of neurotrauma still holds many gaps. As TBI leads to high social costs and personal suffering, strongly impacting the quality of life, understanding and appreciating the progress made so far is an important step. The two stories, one of a construction worker and one of an artist showcase the impact of neurotrauma on people beyond basic functions, as well as its ability to reshape their personalities, abilities, and inner worlds.

“As long as our brain is a mystery, the universe, the reflection of the structure of the brain, will also be a mystery.”

**– Santiago Ramón y Cajal**
